# Midland Railway

**Published:** 1886-01

**Authors:** 


### Midland Railway.

We have recently had the privilege of a trip of 115 miles from Cincinnati to Columbus, Ohio, over a new road and through, to us, an entirely new country, via the Cincinnati, Columbus and Midland Railway, which has been put in operation within the last twelve months. With the exception of the exit from Cincinnati, the route is in a bee line, consequently the distance and time of travel are materially reduced as compared with those of the other lines between the points. It passes through some of the most beautiful country of Ohio—the magnificent grazing counties of Clermont, Clinton, Fayette, Madison, and Franklin. Great care in construction is shown by the smoothness of the road. The facilities afforded have already attracted a large share of both freight and passenger traffic, and we can most heartily recommend it for rapid and easy travel.

